# Few-Shot Learning for Prostate Cancer Detection on MRI: Comparative Analysis with Radiologists’ Performance

**DOI:** 10.1007/s10278-025-01581-9

**Published:** 2025-06-25

**Authors:** Yosuke Yamagishi, Yasutaka Baba, Jun Suzuki, Yoshitaka Okada, Kent Kanao, Masafumi Oyama

**Affiliations:** 1 Department of Diagnostic Radiology, Saitama Medical University International Medical Center, Saitama Medical University International Medical Center, Hidaka, Japan; 2 Department of Urological Oncology, Saitama Medical University International Medical Center, Hidaka, Japan; 3 Department of Radiology and Biomedical Engineering, Graduate School of Medicine, The University of Tokyo, Bunkyo City, Tokyo, Japan

**Keywords:** Prostate cancer, MRI, Few-shot learning, Transformer, Mamba, CNN, ImageNet pretraining

## Abstract

Deep-learning models for prostate cancer detection typically require large datasets, limiting clinical applicability across institutions due to domain shift issues. This study aimed to develop a few-shot learning deep-learning model for prostate cancer detection on multiparametric MRI that requires minimal training data and to compare its diagnostic performance with experienced radiologists. In this retrospective study, we used 99 cases (80 positive, 19 negative) of biopsy-confirmed prostate cancer (2017–2022), with 20 cases for training, 5 for validation, and 74 for testing. A 2D transformer model was trained on T2-weighted, diffusion-weighted, and apparent diffusion coefficient map images. Model predictions were compared with two radiologists using Matthews correlation coefficient (MCC) and F1 score, with 95% confidence intervals (CIs) calculated via bootstrap method. The model achieved an MCC of 0.297 (95% CI: 0.095–0.474) and F1 score of 0.707 (95% CI: 0.598–0.847). Radiologist 1 had an MCC of 0.276 (95% CI: 0.054–0.484) and F1 score of 0.741; Radiologist 2 had an MCC of 0.504 (95% CI: 0.289–0.703) and F1 score of 0.871, showing that the model performance was comparable to Radiologist 1. External validation on the Prostate158 dataset revealed that ImageNet pretraining substantially improved model performance, increasing study-level ROC-AUC from 0.464 to 0.636 and study-level PR-AUC from 0.637 to 0.773 across all architectures. Our findings demonstrate that few-shot deep-learning models can achieve clinically relevant performance when using pretrained transformer architectures, offering a promising approach to address domain shift challenges across institutions.

## Introduction

The development of deep-learning models for prostate cancer detection has progressed rapidly in recent years. Many researchers have constructed high-performance models using large-scale datasets, paving the way for clinical applications [1–6]. In many cases, these models have demonstrated performance comparable to or surpassing that of radiologists [7].

However, in the field of medical image diagnosis, domain shift can occur owing to various factors, such as differences in imaging equipment, patients’ racial backgrounds, and variations in imaging protocols across facilities [8]. This domain shift can cause models that perform well in one environment to underperform significantly in another.

Therefore, it is crucial to fine-tune the models at each facility and adapt them to specific environments. However, collecting cases at medical institutions is often difficult and building large-scale datasets is not easily achievable. Therefore, it is extremely important to perform fine-tuning using a small number of cases. Previous studies have investigated the relationship between the model performance and the amount of training data [7]. However, research directly comparing the model performance under few-shot learning conditions with the diagnostic accuracy of radiologists is limited.

To address these challenges, we propose an enhanced approach that maximizes the utility of limited magnetic resonance images (MRI) datasets. Our method expands upon established methodologies and previous techniques by adopting a 2D model to leverage the multi-slice nature of MRI data [9], effectively increasing the amount of training data per case. We streamlined the data preparation process using an efficient slice-by-slice labeling system based on the biopsy results. Despite working with only 20 cases, our goal was to develop a deep-learning model that achieves diagnostic accuracy comparable to that of experienced radiologists. We investigated the effectiveness of fine-tuning techniques using limited data and explored how these models can be realistically implemented in clinical settings.

Herein, we present a method for each medical facility to efficiently optimize models using their own data, thereby improving the diagnostic accuracy and efficiency. Furthermore, we investigate which model architectures and training strategies perform best in few-shot learning conditions through extensive validation on an external dataset. This comprehensive approach provides a path for solving the problem of domain shift while reducing the costs associated with large-scale data collection and labeling.

By demonstrating that a model can be developed with minimal data from a facility and identifying the optimal architectural choices for few-shot learning, we show that effective model development is possible for each facility, regardless of the domain shift. Our comparative analysis of diverse architectures—from conventional CNNs to state-of-the-art transformer models—offers practical insights for implementing AI solutions with limited data. Consequently, we expect that any facility can develop a model to enhance diagnostic accuracy and efficiency by collecting a small number of cases, selecting appropriate model architectures, and performing simple labeling.

## Materials and Methods

This retrospective study was conducted at a single institution and approved by the Institutional Review Board of our institution. This study adhered to the guidelines outlined in the Checklist for Artificial Intelligence in Medical Imaging (CLAIM) 2024 update [10].

### Dataset

We used multiparametric MRI images comprising T2-weighted images (T2WI), diffusion-weighted images (DWI), and apparent diffusion coefficient (ADC) maps acquired at our hospital between 2017 and 2022. MRI examinations were performed using a Philips Achieva scanner (Philips, Amsterdam, Netherlands) between April 2017 and July 2022.

We randomly selected 100 cases, all of which had undergone MRI-targeted prostate biopsy with a subsequent pathological diagnosis. As a result of the biopsy, cases diagnosed as cancer by pathological diagnosis were considered cancer positive.

**Fig. 1:**
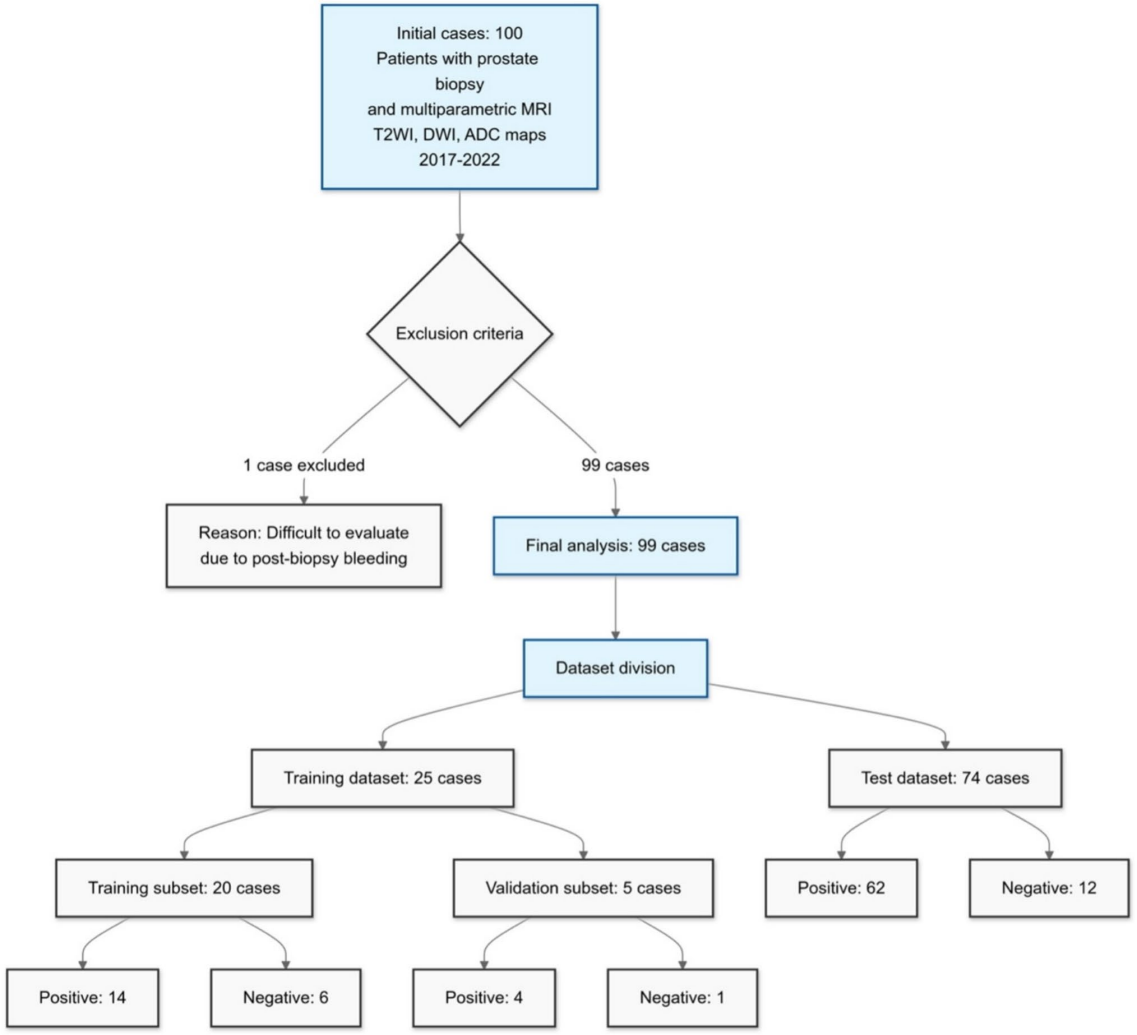
Flowchart of patient inclusion and data distribution in the study of multiparametric MRI data for prostate cancer detection. The flowchart illustrates the selection process from 100 initial cases to 99 analyzed cases, with one patient excluded because of post-biopsy bleeding. This shows the division into training (*n* = 25) and test (*n* = 74) datasets, with further subdivision of the training set into training (*n* = 20) and validation (*n* = 5) subsets. Positive and negative biopsy results are obtained for each patient subset. MRI, magnetic resonance imaging; T2WI, T2-weighted image; DWI, diffusion-weighted image; ADC, apparent diffusion coefficient

One case was excluded because of difficulty in evaluation owing to bleeding from the pre-MRI biopsy. The remaining 99 cases were divided into the training and test datasets. The training dataset was further divided into training and subsets. Figure 1 shows a flowchart of patient selection and data distribution.

### Data Preprocessing

The magnetic resonance (MR) image data varied in resolution, ranging from 224 × 224 to 1008 × 1008 pixels. To standardize the input, all the images were resized to 224 × 224 pixels using bilinear interpolation. The pixel values were normalized to 256 levels (0–255 for each pixel).

The model input consisted of T2WIs, DWIs, and ADC maps stacked in the channel direction as NumPy arrays [11].

### Model Development

**Fig. 2:**
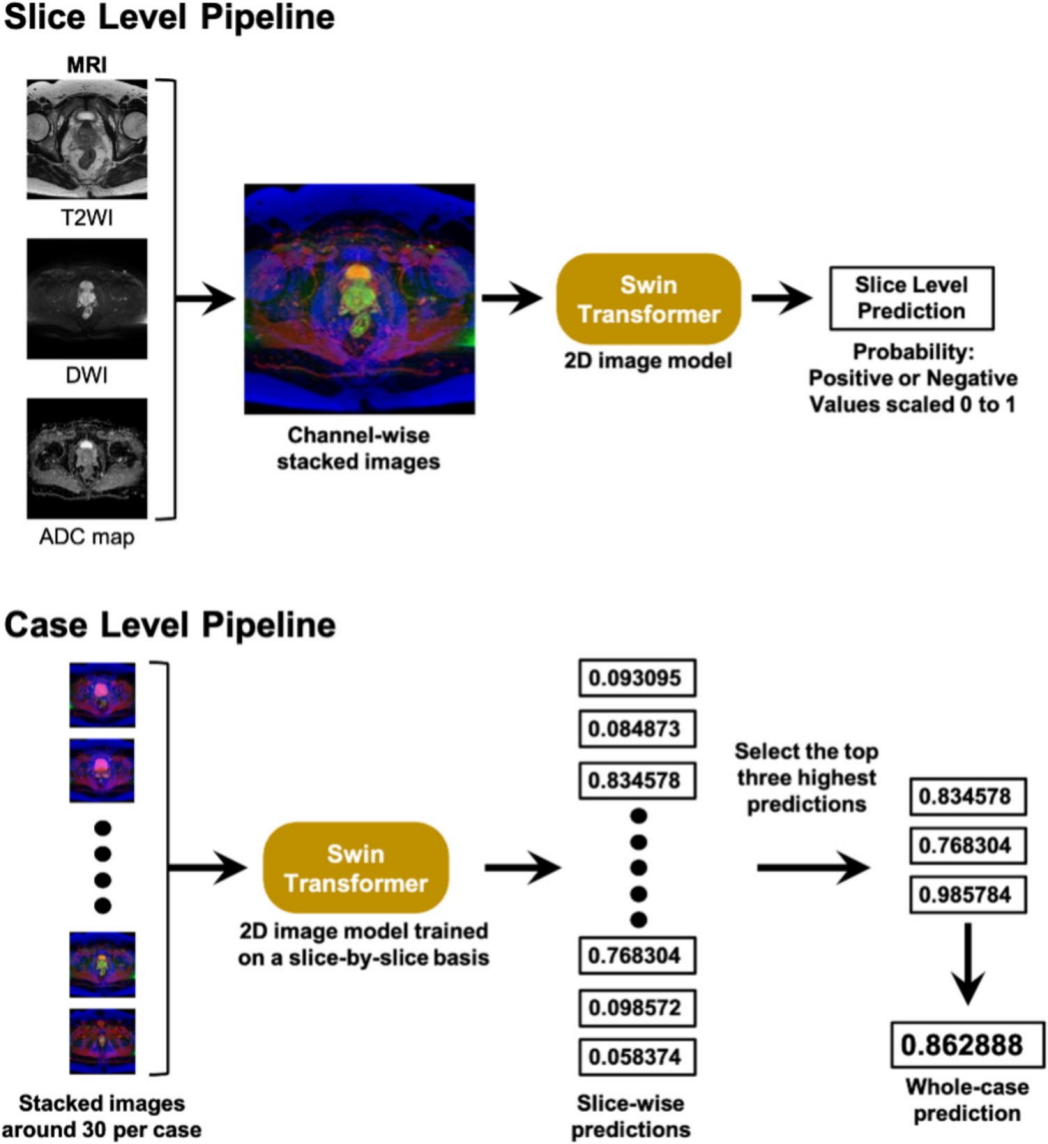
MR image analysis pipelines. This figure presents two interconnected pipelines for magnetic resonance (MR) image analysis: the upper slice-level pipeline for training processes of individual MR image slices through channel-wise stacking, and a Swin Transformer, producing slice-level predictions. The lower case-level prediction aggregates multiple stacked images, processes them through a slice-trained Swin Transformer, and selects the top three predictions to generate a case-level prediction. Both pipelines use the same 2D image models and output probability values from 0 to 1 for positive/negative predictions. MRI, magnetic resonance imaging; T2WI, T2-weighted image; DWI, diffusion-weighted image; ADC, apparent diffusion coefficient

An overview of the proposed approach is shown in Fig. 2.

To train the model with a small number of MR images, we adopted a 2D model instead of 3D models such as 3D convolutional neural network (CNN) models [12], which are frequently used in conventional MR image classification. Using a 2D model, the amount of data input to the model was increased by labeling each slice. The training data were labeled for each slice, depending on the presence or absence of lesions, and 645 2D image data points (96 lesion-positive, 549 lesion-negative) were obtained. The slice-level annotation was performed by a post-graduate year 3 radiology resident using the in-hospital reading system. During this process, the resident referenced pathology reports to ensure accuracy. Even when tumorous lesions were found in cases determined to be negative by pathology, they were appropriately labeled as negative in accordance with the pathology results. Unlike creating detailed segmentation masks, the annotation process only required slice-level binary classification (presence or absence of lesions) while viewing pathology report slices, which significantly reduced the workload burden on the resident.

We used the Swin Transformer Small [13] with a patch size of 4 and an image size of 224. The pre-trained weights of the model were initialized using the ImageNet-22 k dataset [14] and fine-tuned on the ImageNet-1 k dataset [15], which is publicly available in the Timm library [16].

During training, we used a batch size of 8 and trained the model for 10 epochs. The model from the final epoch was selected for evaluation. A separate validation dataset (*n* = 5) was used to verify the slice-level prediction performance of the model. The AdamW optimizer [17] was employed, and a warm-up strategy was applied to gradually increase the learning rate to 0.0001 during the first epoch, followed by gradual decay for the remaining 9 epochs. A binary cross-entropy (BCE) loss function was used.

The Albumentation library [18] was used for data augmentation during the training. The augmentation techniques applied included random resizing and cropping between 85 and 100% of the original scale, random rotation with a maximum angle of 15°, random horizontal and vertical flipping, random hue and saturation shifts, and cutout with a maximum of 5 randomly placed black patches up to 5% of the image size. After applying these data augmentations, normalization was performed using the mean and standard deviation of the RGB values from the ImageNet dataset, and the normalized images were used as inputs to the model.

The values predicted by the model were obtained for each slice. The three highest predicted values for each case were extracted and their average values were calculated as the predicted values for each case.

The experiments were conducted using Python 3.10 and PyTorch 2.0 [19].

### Statistical Analysis

Following the pipeline described in Fig. 2, slice-level predictions from our model were converted to study-level predictions by averaging the top three slice-level prediction values for each case. All evaluations and statistical analyses were performed at the study level, ensuring that the unit of analysis was the case rather than individual slices, thus accounting for the correlation between slices within the same volume.

The performance of the obtained model is evaluated using multiple metrics. The area under the receiver operating characteristic curve (ROC-AUC), precision, recall, and accuracy were calculated. However, to address the challenges of imbalanced datasets, we primarily focused on the Matthews correlation coefficient (MCC) and the F1 score for performance comparisons. All statistical metrics were calculated using the scikit-learn library [20].

The bootstrap method was used to calculate the 95% Credible intervals (CIs) for all metrics. The performance of the model, particularly in terms of F1 score and MCC, was compared with the reading results of two radiologists (hereinafter referred to as Radiologist 1 and Radiologist 2, with 15 and 8 years of experience, respectively) to assess its potential clinical utility using a previously validated method [21]. They used the hospital’s standard clinical reading system to diagnose the presence or absence of cancer in each case, following their normal clinical workflow. The radiologists were instructed to provide binary assessments (cancer present/absent) without Gleason score estimations, as this binary classification matched our model’s output for direct comparison.

### Visualization of Model Attention

In addition, gradient-weighted class activation mapping (GradCAM) [22] was used to visualize the importance of pixels contributing to the prediction of a particular class using the gradient information of the model. The gradient of the model output was calculated and weighted for each channel of the feature map. The weighted feature maps for each channel were then summed to produce an importance map for the prediction results.

### External Dataset Validation

#### Dataset

To evaluate the generalizability of our few-shot learning approach, we conducted external validation using the publicly available Prostate158 dataset [23], which contains multiparametric MRI data with DWI, ADC maps, and T2WI sequences, along with tumor segmentation data. This dataset enabled us to assign slice-level cancer positive and negative labels using the same pipeline as our institutional dataset. Following our few-shot learning paradigm, we allocated 20 cases for training, 10 cases for validation, and the remaining 128 cases for testing (84 positive, 44 negative).

#### Model Architectures

We evaluated nine different model architectures to determine the optimal architecture for few-shot learning in prostate cancer detection. For each architectural category, we selected representative models with approximately 20–50 M parameters to ensure fair comparison across different design paradigms while maintaining computational efficiency.

1. Convolutional Neural Networks (CNNs):
  ⚬ ResNet50d (pretrained on ImageNet-1 K) [24, 25]
  ⚬ EfficientNetV2-Small (EfficientNetV2, pretrained on ImageNet-22 K with fine-tuning on ImageNet-1 K) [26, 27]
  ⚬ ConvNeXtV2-Tiny (ConvNeXtV2, pretrained on ImageNet-22 K and fine-tuned on ImageNet-1 K) [28, 29]
2. Vision Transformers:
  ⚬ ViT-Small Patch16 (ViT-S, pretrained on ImageNet-22 K and fine-tuned on ImageNet-1 K) [30]
  ⚬ DeiT3-Small Patch16 (DeiT-S, pretrained on ImageNet-1 K) [31]
  ⚬ Swin Transformer-Small (Swin-S, pretrained on ImageNet-22 K with multi-scale training and fine-tuned on ImageNet-1 K)
3. Hybrid Architectures:
  ⚬ MaxViT-Tiny (MaxViT, pretrained on ImageNet-1 K) [32]
  ⚬ CAFormer-S18 (CAFormer, pretrained on ImageNet-22 K) [33]
4. Mamba:
  ⚬ MambaVision-Small (Mamba-S, pretrained on ImageNet-1 K, accessed from Hugging Face model hub: nvidia/MambaVision-S-1 K) [34, 35]

All models except MambaVision were implemented using the Timm library with their respective pretrained weights. MambaVision, a state-of-the-art architecture designed for efficient training and improved performance, was accessed through the Hugging Face model repository.

#### Training Methodology

We employed the same data preprocessing, augmentation, and training methodology as described in our main study. Additionally, we investigated the impact of two key factors:

1. Pretraining Influence: We compared models with various pretraining strategies, including models pretrained on ImageNet-1 K and ImageNet-22 K, to assess the impact of pretraining data volume and diversity on few-shot learning performance.
2. Loss Functions: We evaluated both BCE loss and Focal Loss [36] to determine which is more effective for handling the class imbalance inherent in prostate cancer detection.

The training process for all models followed the same protocol as our main study, with batch size 8, learning rate warm-up to 0.0001 during the first epoch followed by gradual decay, and 10 training epochs. Data augmentation techniques remained consistent with our primary methodology.

#### Evaluation Metrics

Given the imbalanced nature of the test set (84 positive, 44 negative), we expanded our evaluation metrics beyond those used in the main study:

1. Study-level Metrics:
  ⚬ ROC-AUC
  ⚬ Area Under the Precision-Recall Curve (PR-AUC), which is particularly valuable for imbalanced datasets
2. Slice-level Metrics:
  ⚬ Slice-level ROC-AUC
  ⚬ Slice-level PR-AUC

This comprehensive evaluation approach allowed us to assess both the overall diagnostic performance of the models and their ability to accurately identify cancer-positive slices.

The external validation was designed to determine whether the few-shot learning approach could maintain comparable performance across different datasets and to identify the most robust model architecture and training strategy for clinical deployment under limited data conditions.

## Results

### Dataset Characteristic

Table 1 shows that all study participants were Asians, with a mean age of 71.39 years (standard deviation [SD] 7.43) overall, while the training group had a mean age of 73.12 years (SD 7.18) and the test group had a mean age of 70.81 years (SD 7.47). Regarding Gleason scores, 19.2% (19/99) of participants had no cancer. It should be noted that all cases were patients who required biopsy due to suspected prostate cancer, resulting in this high cancer detection rate (80.8%). In the training group, Gleason score 8 (4 + 4) was most common at 44.0% (11/25), while the test group showed a more balanced distribution across scores from 6 to 10, with Gleason 7 being most frequent (35.1% combined for 3 + 4 and 4 + 3).

**Table 1:** Demographic characteristics of the study participants overall and by group allocation

Characteristic	Overall (N = 99)	Training Group (N = 25)	Test Group (N = 74)
Age, years, mean ± SD	71.39 ± 7.43	73.12 ± 7.18	70.81 ± 7.47
Race, *n* (%)			
Asian	99 (100%)	25 (100%)	74 (100%)
Gleason Score			
No cancer	19 (19.2%)	7 (28.0%)	12 (16.2%)
3 + 3 = 6	7 (7.1%)	0 (0%)	7 (9.5%)
3 + 4 = 7	17 (17.2%)	3 (12.0%)	14 (18.9%)
4 + 3 = 7	13 (13.1%)	1 (4.0%)	12 (16.2%)
4 + 4 = 8	22 (22.2%)	11 (44.0%)	11 (14.9%)
4 + 5 = 9	16 (16.2%)	3 (12.0%)	13 (17.6%)
5 + 4 = 9	4 (4.0%)	0 (0%)	4 (5.4%)
5 + 5 = 10	1 (1.0%)	0 (0%)	1 (1.4%)

The actual imaging parameters achieved across all examinations were as follows: slice thickness was consistently maintained at 3.0 mm for all sequence types. The detailed parameters obtained for each sequence are summarized in Table 2, including TR, TE, field of view, and matrix size.

**Table 2:** Distribution of MRI studies and imaging parameters for training and test datasets: number of studies by tesla strength (upper) and mean imaging parameters with ranges (lower)

	Training Case	Test Case
3 Tesla studies	17	55
1.5 Tesla studies	8	19
Parameter		
T2WI TR (ms)	5612 (3500–6550)	5672 (4048–7604)
DWI/ADC map TR (ms)	6332 (6331–7500)	6270 (5750–7812)
T2WI TE (ms)	96.8 (90.0–100.0)	97.6 (90.0–100.0)
DWI/ADC map TE (ms)	78.7 (74.7–83.0)	77.8 (74.4–87.3)

*T2WI*, T2-weighted image; *TR*, repetition time; *DWI*, diffusion-weighted image; *ADC*, apparent diffusion coefficient; *TE*, echo time

### Model Performance and Comparison with Radiologists

For the validation data, the model achieved a ROC-AUC of 0.690 and PR-AUC of 0.358 at the slice-level.

The constructed model for prostate cancer classification demonstrated a significant predictive capability at the study-level on the test dataset. Its performance metrics were as follows: accuracy, 0.611 (95% CI: 0.500 to 0.723); precision, 0.947 (0.865 to 1.00); recall, 0.567 (0.439 to 0.690); ROC-AUC, 0.730 (0.588 to 0.847); MCC, 0.297 (0.095 to 0.474); and F1 score, 0.707 (0.598 to 0.847).

For comparison, we evaluated the performance of the two radiologists in the same cases. Radiologist 1 achieved an accuracy of 0.641 (0.528–0.750), precision of 0.929 (0.840–1.00), recall of 0.619 (0.492–0.742), MCC of 0.276 (0.054–0.484), and F1 score of 0.741 (0.632–0.832). Radiologist 2 showed a higher performance level, with an accuracy of 0.803 (0.708–0.889), precision of 0.961 (0.898–1.00), recall of 0.797 (0.698–0.889), MCC of 0.504 (0.289–0.703), and F1 score of 0.871 (0.800–0.931).

To compare the performance of the model with that of radiologists, we focused on the differences in the MCC and F1 scores. When compared to Radiologist 1, the model showed a slightly higher MCC (difference of 0.021, 95% CI: − 0.270 to 0.306) and a slightly lower F1 score (difference of − 0.034, 95% CI: − 0.153 to 0.078), but there were no statistically significant differences. In comparison with Radiologist 2, the MCC of the model was lower, but not significantly (difference of − 0.207, 95% CI: − 0.480 to 0.060), while its F1 score was significantly lower (difference of − 0.16, 95% CI: − 0.287 to − 0.061).

**Fig. 3:**
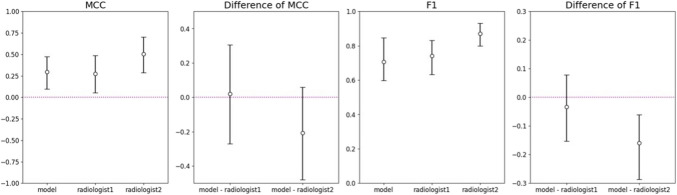
Comparison of MCC and F1 scores between the few-shot learning model and two radiologists for prostate cancer detection, showing absolute values (left panels) and differences between the model and each radiologist (right panels); error bars representing 95% confidence intervals. MCC, Matthews correlation coefficient

The results are summarized in Table 3 and Fig. 3, which provide a comprehensive comparison of the performance metrics between the constructed model and the two radiologists. The analysis suggests that while the model’s performance was comparable to that of Radiologist 1, there is room for improvement to match the higher performance level demonstrated by Radiologist 2, particularly in terms of overall accuracy and recall.

**Table 3:** Comparison of performance metrics between the constructed model and two radiologists (95% Confidence intervals in parentheses). ROC-AUC was not applicable for radiologists as they provided binary predictions rather than probability scores

Metrics	Model	Radiologist 1	Radiologist 2
Accuracy	0.611 (0.500–0.723)	0.641 (0.528–0.750)	0.803 (0.708–0.889)
Precision	0.947 (0.865–1.00)	0.929 (0.840–1.00)	0.961 (0.898–1.00)
Recall	0.567 (0.439–0.690)	0.619 (0.492–0.742)	0.797 (0.698–0.889)
MCC	0.297 (0.095–0.474)	0.276 (0.054–0.484)	0.504 (0.289–0.703)
F1	0.707 (0.598–0.847)	0.741 (0.632–0.832)	0.871 (0.800–0.931)
ROC-AUC	0.723 (0.588–0.847)	Not applicable	Not applicable
PR-AUC	0.939 (0.886-0.978)	Not applicable	Not applicable

*MCC*, Matthews correlation coefficient. ROC-AUC: area under the receiver operating characteristic curve

### Visualization of Model Attention

**Fig. 4:**
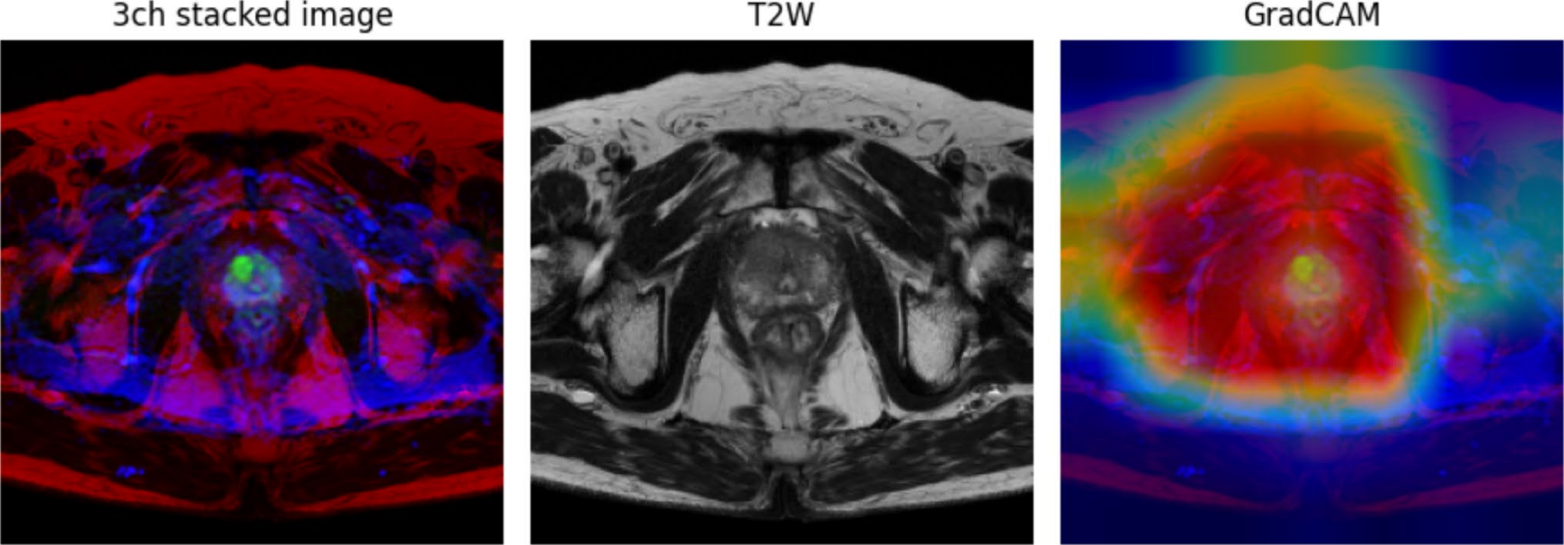
Visualization of prostate MRI analysis, showing (left) the model input as a 3-channel (3ch) stacked image combining T2-weighted images (T2WIs), diffusion-weighted images (DWI), and apparent diffusion coefficient maps (ADC); (center) the original T2WI used for gradient-weighted class activation mapping (GradCAM) overlay; and (right) the resulting GradCAM heatmap highlighting areas of model focus for cancer detection

Figure 4 shows the attention visualization of the model using Grad-CAM for prostate cancer classification. The figure shows a 3-channel stacked image, T2WI, and the corresponding GradCAM visualization.

The visualization demonstrated that the model focused primarily on the entire prostate gland, as indicated by the warm colors (red and yellow) in GradCAM images. The areas of highest attention aligned with the zonal anatomy of the prostate were visible in the T2-weighted image, suggesting that the model recognizes clinically relevant structures. The surrounding tissues, shown in cooler colors (blue and green), received less attention from the model. This indicates that the model appropriately prioritizes the prostate region in its analysis.

### External Dataset Validation Results

The external validation using the Prostate158 dataset revealed substantial differences in model performance under few-shot learning conditions. ImageNet pretraining emerged as a critical factor in model performance across all architectures evaluated. Models trained with ImageNet pretraining consistently outperformed their non-pretrained counterparts across all metrics. For study-level classification, the average ROC-AUC improved from 0.464 (no pretraining) to 0.636 (with pretraining) when using standard BCE loss. Similarly, study-level PR-AUC increased from 0.637 to 0.773. This performance difference was also observed at the slice level, with improvements in both slice ROC-AUC and slice PR-AUC metrics when comparing non-pretrained to pretrained models.

The choice between BCE loss and Focal Loss showed variable effects depending on the model architecture and pretraining status. For pretrained models, Focal Loss generally maintained or slightly improved performance for transformer-based architectures (ViT-S, DeiT-S), while occasionally decreasing performance for CNN-based models. With pretrained models using Focal Loss, DeiT-S achieved high performance with study ROC-AUC of 0.781 and study PR-AUC of 0.893, whereas ViT-S demonstrated strong slice-level metrics with ROC-AUC of 0.847 and PR-AUC of 0.602.

Among the nine architectures evaluated, transformer-based models generally outperformed conventional CNN architectures in the few-shot learning scenario. Swin Transformer-Small demonstrated the highest overall performance with ImageNet pretraining and BCE loss, achieving a study ROC-AUC of 0.802 and PR-AUC of 0.880. However, its performance dramatically decreased when using Focal Loss (study ROC-AUC: 0.535, study PR-AUC: 0.705). DeiT-S showed consistently strong performance with pretraining, particularly maintaining high metrics when using Focal Loss compared to other architectures. MaxViT-Tiny showed moderate performance with pretraining (study ROC-AUC: 0.733, study PR-AUC: 0.860), demonstrating the value of hybrid architectures.

The state-of-the-art MambaVision-S model showed comparable performance to traditional transformer models when pretrained (study ROC-AUC: 0.729, PR-AUC: 0.846), though it did not outperform established architectures like Swin-S or DeiT-S in this specific few-shot learning task. Conventional CNN architectures such as ConvNeXtV2-Tiny demonstrated substantially lower maximum performance (study ROC-AUC: 0.483), highlighting their limitations in few-shot learning scenarios.

When comparing model categories, transformer-based architectures (average ROC-AUC: 0.769) significantly outperformed CNN-based models (average ROC-AUC: 0.506) and showed better performance than hybrid architectures (average ROC-AUC: 0.590) and Mamba-based models (ROC-AUC: 0.729) with ImageNet pretraining and BCE loss.

**Fig. 5:**
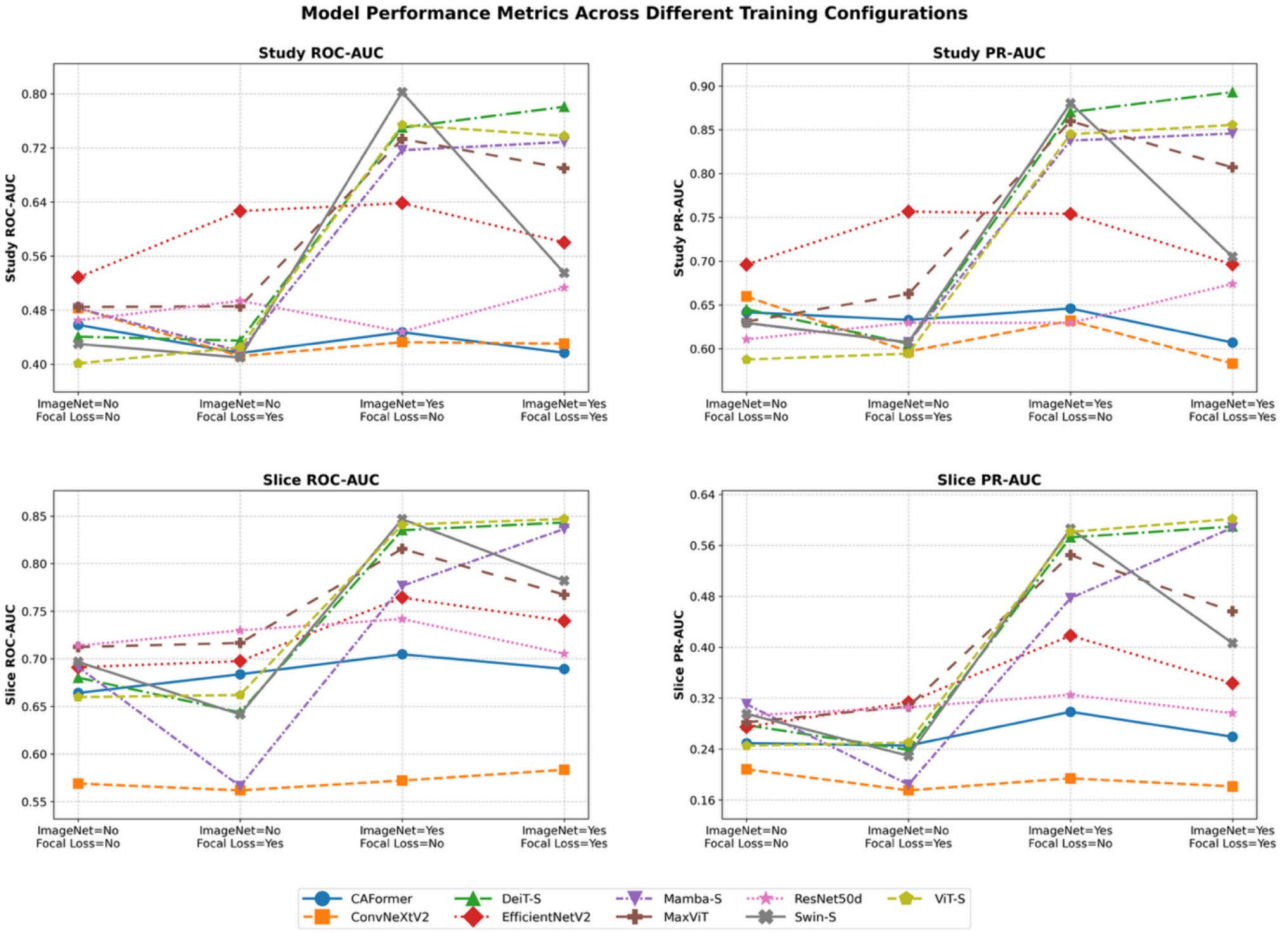
Performance metrics of nine deep learning architectures on the Prostate158 external validation dataset under few-shot learning conditions. The graphs show study-level and slice-level ROC-AUC and PR-AUC across four training configurations: with/without ImageNet pretraining and with/without Focal Loss. Different model architectures are represented by colored lines as indicated in the legend

Figure 5 illustrates the comparative performance of all nine models across different training configurations, highlighting the consistent superiority of transformer-based architectures with ImageNet pretraining. Overall performance results are detailed in Table 4.

**Table 4:** Performance metrics of different model architectures on the Prostate158 external validation dataset under few-shot learning conditions. For each model, results are shown across four training configurations: with/without ImageNet pretraining and with/without Focal Loss. The highest values for each metric are highlighted in bold, and the second highest by an underline. Study-level metrics evaluate the model's ability to classify entire cases, while slice-level metrics assess detection performance on individual MRI slices

Model	Pretrained	Focal Loss	Study ROC-AUC	Study PR-AUC	Slice ROC-AUC	Slice PR-AUC
ResNet50d	No	No	0.465	0.611	0.714	0.293
	No	Yes	0.494	0.630	0.730	0.305
	Yes	No	0.448	0.629	0.742	0.325
	Yes	Yes	0.513	0.674	0.705	0.296
EfficientNetV2-Small	No	No	0.528	0.696	0.691	0.275
	No	Yes	0.626	0.757	0.698	0.313
	Yes	No	0.638	0.754	0.764	0.418
	Yes	Yes	0.580	0.696	0.740	0.343
ConvNeXtV2-Tiny	No	No	0.483	0.660	0.569	0.208
	No	Yes	0.412	0.597	0.562	0.175
	Yes	No	0.433	0.632	0.572	0.194
	Yes	Yes	0.430	0.583	0.583	0.181
ViT-Small Patch16	No	No	0.401	0.588	0.660	0.245
	No	Yes	0.425	0.594	0.662	0.250
	Yes	No	0.754	0.845	0.841	0.581
	Yes	Yes	0.738	0.856	**0.847**	**0.602**
DeiT3-Small Patch16	No	No	0.441	0.645	0.680	0.278
	No	Yes	0.435	0.606	0.644	0.239
	Yes	No	0.750	0.870	0.835	0.573
	Yes	Yes	0.781	**0.893**	0.843	0.590
Swin Transformer-Small	No	No	0.430	0.629	0.697	0.295
	No	Yes	0.410	0.607	0.642	0.229
	Yes	No	**0.803**	0.880	**0.847**	0.586
	Yes	Yes	0.535	0.705	0.782	0.407
MaxViT-Tiny	No	No	0.485	0.631	0.712	0.282
	No	Yes	0.485	0.663	0.717	0.307
	Yes	No	0.733	0.860	0.816	0.545
	Yes	Yes	0.690	0.807	0.767	0.456
CAFormer-S18	No	No	0.458	0.642	0.664	0.249
	No	Yes	0.416	0.633	0.684	0.245
	Yes	No	0.447	0.646	0.705	0.298
	Yes	Yes	0.417	0.607	0.689	0.259
MambaVision-Small	No	No	0.483	0.629	0.691	0.310
	No	Yes	0.419	0.607	0.566	0.184
	Yes	No	0.717	0.838	0.777	0.477
	Yes	Yes	0.729	0.846	0.836	0.588

*ROC-AUC*, area under the receiver operating characteristic curve. *PR-AUC*, area under the precision-recall curve

## Discussion

We developed a deep-learning model to classify prostate magnetic resonance images (MRI) based on T2-weighted images (T2WI), diffusion-weighted images (DWI), and apparent diffusion coefficient (ADC) maps to determine whether a biopsy is positive or negative. We devised an algorithm that divides MR images into slice-level sequences and stacks them together, enabling analysis using a two-dimensional model.

Despite training on only 20 cases, the model achieved a classification performance comparable to that of radiologists. The data used in this study consisted of cases in which biopsy was deemed necessary because of remaining suspicion of malignancy based on radiological findings. Many of these cases were difficult to distinguish as benign or malignant; therefore, the results obtained were significantly valuable.

Our external validation on the Prostate158 dataset offered important insights into deep learning architecture performance under few-shot conditions. ImageNet pretraining emerged as the critical factor, with pretrained models outperforming non-pretrained counterparts by approximately 0.172 in ROC-AUC and 0.136 in PR-AUC. Transformer-based architectures (particularly DeiT-S, ViT-S, and Swin-S) consistently outperformed conventional CNNs, suggesting their self-attention mechanisms better leverage limited training data. The state-of-the-art MambaVision model performed comparably to established transformers without demonstrating significant advantages. These findings emphasize that pretrained transformer-based models offer the most promising approach for few-shot learning scenarios in addressing domain shift challenges, providing practical guidance for clinical implementation with minimal data requirements.

Several deep-learning models have been proposed for the classification of csPCa images. Aldoj and colleagues developed a highly accurate 3D CNN model with an ROC-AUC of 0.897 [37]. However, their model required MRI data from a sizable cohort of 175 patients for training purposes. Chen et al. utilized a 2D CNN pretrained on the ImageNet dataset to achieve an AUC of 0.83 [38]. They performed training using 330 samples from the PROSTATEx dataset [39]. In our study, since there was only one negative case at the study-level in our validation data, we evaluated a 2D-based approach instead. Although it is challenging to directly compare performances because of differences in the target populations, we achieved promising results, with performance comparable to that of a radiologist using only 20 cases.

The main objective of this study was to propose a solution for domain shift [8], which can significantly reduce the accuracy, even for highly accurate models trained on large data sets, because of variations in imaging conditions and imaging equipment. The development of models with sufficient performance, based on a small number of cases at each facility, will enable individual facility-specific model development without considering the domain shift. Furthermore, by utilizing only cases from the facility itself, many issues related to data confidentiality can also be resolved. Here, we developed a high-performance model based on a small number of cases, and established a pathway to resolve these issues.

The future direction of artificial intelligence (AI) in medical imaging is increasingly turning toward the use of foundational models. For instance, BiomedCLIP trained on 15 million text-image pairs from the open PMC dataset has shown remarkable performance in radiology [40]. Similarly, CT-CLIP, aimed at becoming a foundational model for lung computed tomography, was developed using scans from more than 20,000 patients and has achieved impressive results [41]. The introduction of TotalSegmentator MRI has enabled segmentation of virtually every organ in the body [42]. These advancements suggest that the emergence of foundational models or their fine-tuning may lead to the development of AI systems that rely on radiologists’ expertise.

There are only a few reported studies on few-shot learning using MRI. Dhinagar et al. [43] proposed few-shot learning for classifying autism spectrum disorder from MR images. In their approach, they first trained a 3D CNN model using the Autism Brain Imaging Data Exchange dataset [44] and then fine-tuned 20 cases for each site. There are many similarities between their research and ours, such as the use of a small number of data points for fine-tuning (20 cases) and the focus on analyzing the MRI data. We anticipate that performance comparisons between AI and physicians, such as radiologists, under few-shot learning conditions, will become increasingly important. Our research laid the groundwork for this crucial line of inquiry.

Our study had several limitations. First, although we conducted external validation on the Prostate158 dataset, our primary model’s performance was initially evaluated at a single institution, potentially limiting its generalizability. The external validation provided valuable insights into architecture selection and pretraining strategies, but further evaluation across multiple institutions with diverse imaging protocols would strengthen our findings. Second, we did not validate whether combining our model with radiologists would improve diagnostic accuracy in clinical settings. The proposed algorithm determines whether each slice contains malignant findings, enabling the presentation of noteworthy slices, which could potentially enhance radiologists’ performance. Third, our evaluation focused on cases where biopsies were performed due to suspicious findings, excluding cases without malignancy indicators. To develop a comprehensive model applicable to all cases, including those where biopsies are unnecessary, alternative labeling strategies should be considered. Finally, while our external validation compared multiple architectures, further investigation into domain adaptation techniques could further improve performance when transferring models between institutions.

In conclusion, we developed a slice-based MRI model capable of predicting malignancy in biopsies using only 20 cases, with performance comparable to an experienced radiologist. Our external validation demonstrated that pretrained transformer-based architectures consistently outperform conventional CNNs in few-shot learning scenarios, with ImageNet pretraining emerging as a critical factor for success. This approach significantly reduces annotation costs and resource requirements for model development while addressing domain shift challenges that typically hinder AI deployment across institutions. The finding that a model trained on just 20 cases can achieve clinically relevant performance has important implications for practical implementation at facilities with limited data resources. Future research should explore applications to other diseases, extend beyond classification to segmentation and object detection tasks, and investigate domain adaptation techniques to further enhance cross-institutional generalizability.

## Data Availability

The clinical data analyzed in this study from our institution are not publicly available due to patient privacy and institutional ethics regulations. Access to these data would require appropriate ethics approval and data sharing agreements with the participating institution. The external dataset used in this study (Prostate158) is publicly available and can be accessed following the instructions provided at https://github.com/kbressem/prostate158. This dataset was used under the terms and conditions specified by the original authors.

## Abbreviations

ADC: Apparent diffusion coefficient
AI: Artificial intelligence
CI: Confidence intervals
CNN: Convolutional neural network
DWI: Diffusion-weighted images
GradCAM: Gradient-weighted class activation mapping
T2WI: T2-weighted images
MCC: Matthews correlation coefficient
